# Riders’ Effects on Horses—Biomechanical Principles with Examples from the Literature

**DOI:** 10.3390/ani13243854

**Published:** 2023-12-15

**Authors:** Hilary Mary Clayton, Russell MacKechnie-Guire, Sarah Jane Hobbs

**Affiliations:** 1 Large Animal Clinical Sciences, College of Veterinary Medicine, Michigan State University, East Lansing, MI 48824, USA; 2 Equine Department, Hartpury University, Hartpury House, Gloucester, Gloucestershire GL19 3BE, UK; russell.mackechnie-guire@hartpury.ac.uk; 3 Research Centre for Applied Sport, Physical Activity and Performance, University of Central Lancashire, Preston PR1 2HE, UK; sjhobbs1@uclan.ac.uk

**Keywords:** equestrian, biomechanics, gravitational effects, inertial effects, harmonics, harmony, horse–rider interaction

## Abstract

**Simple Summary:**

Equestrian sports include a diverse range of activities requiring a wide variety of technical skills, all of which are based on collaboration between a rider and a horse. During training, horses are taught to respond appropriately to the rider’s cues or “aids”. In addition to these trained responses, the rider’s weight is associated with mechanical effects that are based on physical principles governing the behavior of, and interactions between, bodies in the environment. The effects of gravity, inertia, and turning have predictable effects on the horse, but these may be modified by the rider’s symmetry, balance, and posture. The ultimate goal is for the rider to have a harmonious relationship with the horse, which is addressed in the final section of this article exploring the biomechanical underpinnings of the harmony between riders and horses.

**Abstract:**

Movements of the horse and rider in equestrian sports are governed by the laws of physics. An understanding of these physical principles is a prerequisite to designing and interpreting biomechanical studies of equestrian sports. This article explains and explores the biomechanical effects between riders and horses, including gravitational and inertial forces, turning effects, and characteristics of rider technique that foster synchronous movement with the horse. Rider symmetry, posture, and balance are discussed in the context of their relationship to rider skill level and their effects on the horse. Evidence is presented to support the feasibility of improving equestrian performance by off-horse testing followed by unmounted therapy and exercises to target the identified deficiencies. The elusive quality of harmony, which is key to a true partnership between riders and horses, is explored and described in biomechanical terms.

## 1. Introduction

Equestrian sports encompass a diverse range of activities, each with specific requirements for speed, strength, and agility. This article focusses on the general skills needed by the rider to communicate effectively with the horse using illustrations from a range of sports disciplines, including racing, jumping, and dressage. The rider actively influences the horse’s performance by applying aids that elicit a trained response. These aids are based on changes in pressure applied to specific areas of the horse’s body by the rider. The rider’s weight, weight distribution, and movements have a mechanical effect on the horse that is governed by physical laws studied under the general topic of biomechanics, the branch of physics that deals with living organisms [1]. Biomechanical principles can be used to evaluate how gravity, inertia, and turning affect the rider’s interaction with the horse in different gaits. Superimposed on these basic interactions are the effects of the individual rider’s symmetry, balance, and posture that may reflect strategies to improve the rider’s comfort, reduce energy expenditure, or comply with competition rules. On the other hand, riders may inadvertently adopt suboptimal techniques due to their inherent laterality patterns, pre-existing injuries, or poor coaching [1].

Biomechanics and energetics are inextricably linked in equestrian sports. The neuromotor control system is programmed for efficiency in energy expenditure. Sporting activities, on the other hand, often require greater physical effort and energy expenditure than is strictly necessary, such as travelling faster than the energetically optimal speed in racing, moving with great impulsion in dressage, or propelling the body into the air when jumping.

This article focusses on how the rider interacts with the horse within the context of the biomechanical principles that govern these interactions. The goal is to enhance the reader’s understanding of biomechanics in relation to equestrian sports and illustrate how this knowledge can be applied to improve the performance of the rider and horse. This is not intended to be a comprehensive literature review but will draw on published works to illustrate specific principles and applications.

## 2. Biomechanical Effects of the Rider

### 2.1. Effects of Rider’s Weight

The rider’s body is subjected to the influence of gravity, which is constantly pulling the body downward. When mounted, the rider’s weight is the force that presses downward against the saddle and is supported by a reaction force acting in an upward direction through the saddle (saddle reaction force) and the stirrups (stirrup reaction force).

**Figure 1:**
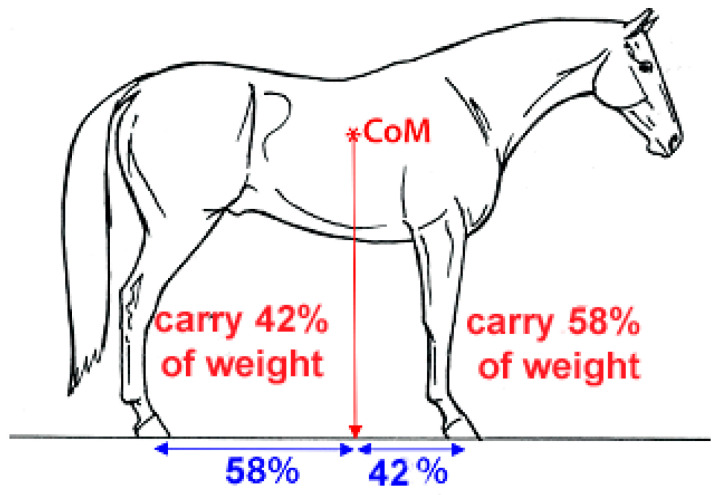
The line of gravity drops vertically from the center of mass (CoM) to the ground. The percentages of the base of support in the cranial and caudal direction to the line of gravity indicate the percentages of body weight carried by the hind limbs and forelimbs, respectively.

When seated in the saddle, the rider’s center of mass (CoM) should be almost vertically above the horse’s CoM, which is approximately at the level of T14 [2]. A vertical line from the horse’s CoM contacts the ground at about 58% of the length of the base of support from the hind limbs towards the forelimbs, indicating that the forelimbs carry 58% of the horse’s weight and the hind limbs carry 42% [3], as illustrated in Figure 1. The presence of a rider increases forelimb loading as shown by the increased fetlock extension [4] and higher vertical GRFs [5]. 

The addition of live weight or dead weight to the horse’s back is associated with increased flexion of the lumbosacral joint [6], causing the lumbar spine to slope ventrally from caudal to cranial direction. Hollowing of the thoracolumbar spine increases with the magnitude of the load [7]. The rider’s effect can be likened to a loaded bookshelf: the heavier the rider and/or the longer the horse’s back, the more likely it is to sag in the middle. The third lumbar vertebra and the sacral tuberosity show smaller vertical excursions when ridden compared to trotting in hand [7] and are associated with greater extension of the thoracolumbar spine compared with unloaded trotting.

Movements of the thoracolumbar intervertebral joints may be induced actively by the concentric contraction of the long, mobilizing back muscles or passively by gravitational, inertial, and ground reaction forces. In the thoracolumbar region, a bilateral concentric action of the epaxial muscles extends the inter-vertebral joints, which has the overall effect of hollowing the back. However, these muscles can also act eccentrically to control the amount of spinal flexion during locomotion [8]. The abdominal and sublumbar muscles act antagonistically to the epaxial muscles. They flex the intervertebral joints in a concentric contraction or limit spinal extension when acting eccentrically. 

There is a debate as to the amount of weight a horse should be expected to carry during a ridden exercise. This is affected by many variables, including speed, terrain, and environmental conditions, in addition to the rider’s weight. Combining data from biomechanical and physiological studies, there is a general consensus that horses being ridden at moderate speeds do not show changes in gait or energetics until the rider/horse body weight ratio (BWR) exceeds 20–25% [9,10,11,12,13]. Some horses may perform safely carrying a higher BWR depending on their size, conformation, and fitness. Icelandic horses and Taishuh ponies reportedly have a higher weight-carrying capacity on a body weight basis than larger horses [9,10,11].

### 2.2. Rider’s Interaction with the Saddle

When riding on the flat, as in dressage, the rider should sit vertically in the middle of the saddle. Judged scores for general impression during a dressage test are influenced by the rider’s verticality and symmetry [14]. The saddle should be a suitable shape and size fit for the rider. If the head plate is too narrow for the horse, the pommel pitches upward, pushing the rider’s seat back on the cantle. Conversely, when the head plate is too wide, the pommel pitches downward and the rider’s seat slides forward [15]. Factors related to saddle design that have been shown to affect rider posture and effectiveness in a manner that was interpreted as being beneficial include having a deformable thigh block that allows greater contact area with more pressure of the rider’s seat against the saddle and a more upright rider position [16]. A saddle designed without flaps has been shown to significantly improve rider stability in all gaits, especially in the transverse direction [17].

When negotiating a turn or circle, the rider’s momentum is directed along the tangent of the circle. The horse’s heading direction changes continuously as it moves around the circle circumference and the inward acceleration tends to cause the rider to slip toward the outside. Riders are encouraged to lower the inside knee and press the inner thigh against the saddle to avoid sliding to the outside. On thoroughbred racetracks with particularly tight turns, jockeys may choose to ride with the outside stirrup shorter than the inside one to combat the tendency to slide to the outside [18].

Friction, which is defined as a force that resists sliding of one surface across another, helps to resist the rider’s tendency to slide across the saddle. The resistance to sliding depends on the coefficient of friction, which is specific to the materials of the two contacting surfaces and the normal (perpendicular) force between them. A larger coefficient of static friction must be overcome to initiate sliding, and then a smaller coefficient of sliding friction resists sliding after the motion has been initiated. The normal force squeezes the two surfaces together, making it more difficult for sliding to occur. For example, when a rider grips with their knees, it increases the normal force between the knees and the saddle. The resulting increase in friction helps to stabilize the rider’s position. 

For sports in which the rider sits in the saddle, such as dressage, the parts of the breeches that are in direct contact with the saddle may be made of, or reinforced by, a material that has a high frictional coefficient with leather, such as silicone. In sports that require rider mobility, such as jumping, it is advantageous to be able to slide over the saddle; thus, the high friction materials in these sports are confined to the knee patches.

### 2.3. Rider’s Pelvic Rotations

The rider’s pelvic movements are key to following the motion of the horse’s back [19]. Rotational movements of the pelvis are pitch, roll, and yaw [1]. 

Pitching motion is rotation around the transverse axis that passes horizontally from left to right. Posterior pelvic pitch (tilt) rotates the tubera coxae backward and down while the front of the pelvis tilts upward, and the rider rocks onto the back of the tubera ischii. This movement flattens the rider’s back (kyphosis) and tucks the seat under the body. Anterior pelvic pitch is forward rotation of the tubera coxae that hollows the rider’s back (lordosis), and the rider rotates onto the front of the tubera ischii. 

Pelvic roll is rotation around the antero-posterior axis; left roll lowers the left side of the pelvis; and right roll lowers the right side.

Pelvic yaw is rotation around the vertical axis; left yaw rotates the left side of the pelvis back; and right yaw rotates the right side of the pelvis back.

The male and female pelvis are shaped differently, which affects the rider’s position and stability. The female pelvis is shorter and broader with more width between the tubera ischii and the aectabulae compared to the male pelvis. As a result, the female rider has a wider base of support on the saddle and shows a greater roll of the pelvis and trunk than the male rider [19]. The female iliac bones have a greater slope than those of males, which is associated with a greater posterior pelvic pitch and a more kyphotic posture during riding [19].

### 2.4. Rider’s Symmetry and Balance

Structural and functional asymmetries between paired structures on the left and right sides of the body are frequently present in riders [20,21,22]. Some of these are consequences of inherent laterality but others have been acquired during riding [20]. A study of 127 riders did not find a leg length discrepancy [20], but this differs from a smaller study that reported a significant leg length inequality based on 17 riders [21]. Further research is needed in this area. Right-handed dressage riders show a greater variation in maximal and mean rein tension for the right rein/hand compared to the left. This suggests that the non-dominant hand is more stable, while the dominant hand is more mobile and provides more rein cues to the horse [23]. 

Despite their inherent asymmetry patterns, riders are usually encouraged to sit and ride symmetrically and to teach the horse to move and load the left and right limbs equally. The rider’s pelvis is particularly important for effective communication with the horse and for applying subtle weight cues [19]. However, a greater pelvic asymmetry has been reported in riders who have ridden for a longer time [24]. The situation is complicated by the fact that both riders and horses have their own laterality pattern. When the horse is moving, the rider needs to withstand the locomotor forces with controlled suppleness in the lumbosacral region and hip joints to allow the pelvis to follow the translations and rotations of the horse’s back and accommodate changes in the back contours whilst maintaining dynamic balance and alignment of the core segments.

Several studies have contributed to the body of knowledge describing the position and common asymmetries in the position and kinematics of riders’ arms and legs. Riders have been reported to show a left shoulder yaw and a greater range of movement in the right shoulder in sitting trot [22]. Novice riders have a larger range of motion of their left elbows and left knees together with a more anterior head tilt than professionals [25]. Mean hip external rotation ranged from 1 to 27° in a group of riders, with 83% of them showing greater external rotation of the right hip [26].

If one of the rider’s body segments is misaligned, the remaining segments compensate to stabilize the rider. For example, a rider who collapses through one hip usually adducts the ipsilateral thigh/knee to stabilize the body, especially when circling. One study found an average of 19° rotation to the right on the right circle and 14° rotation to the left on the left circle [27]. 

**Figure 2:**
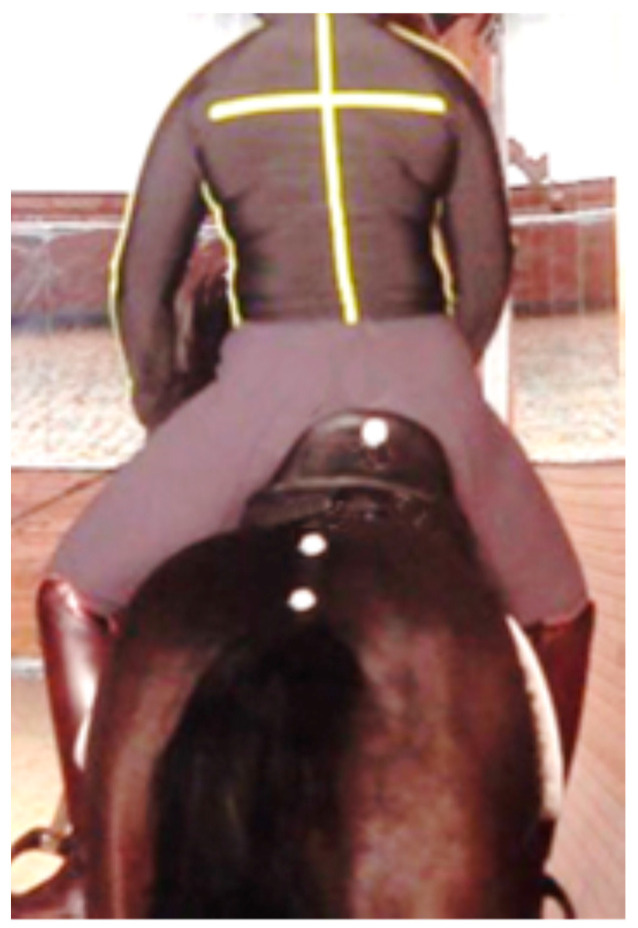
The saddle has slipped markedly to the right, as shown by the position of the white mark on the center of the cantle compared with the white marks on the tail head and lumbar spine of the horse. The rider is attempting to compensate by leaning the shoulders to the left.

From an evidence-based perspective, the primary cause of saddle slip is lameness [28] or movement asymmetry [29] of the horse rather than the rider. Only 21% of horses ridden by crooked riders showed saddle slip, and 16% of riders who collapsed through their left hip had saddle slip to the right. When the saddle slipped to the right, the rider slid to the right and developed concavity of the left side of the body [29] (Figure 2). 

Not surprisingly, asymmetrical rider posture has been related to asymmetrical saddle forces. During sitting trot, if the rider collapses a side of their hip, the force on the horse’s back increases on the contralateral side. If the rider tilts their body to one side, the saddle force increases on the side to which the shoulders are tilted [30].

**Figure 3:**
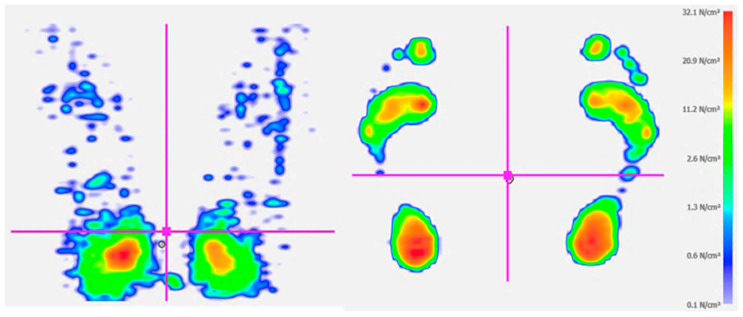
Pressure scans of a rider sitting on a force mat with higher pressure on the left tuber ischium (**left image**) and standing on a pressure mat with higher pressure on the right foot (**right image**). The pressure scans are color coded according to the graduated scale (N/cm^2^) to the right.

In order to better understand rider asymmetries, some studies have investigated functional rider movement patterns off the horse [24,31,32]. To evaluate the rider’s perception of relative weight on the left and right ischial tuberosities, a group of riders sitting on a flat surface were instructed to distribute their weight equally on the left and right sides. The results showed an overall preference to put more weight on the left side [31]. When standing on a force platform, 70% of riders loaded their right foot more than the left [31] (Figure 3).

In comparison, when riding at walk, trot, and canter in a straight line, no differences were found between left and right stirrup forces in eight right-leg dominant riders [23]. When rider asymmetry was mechanically induced by shortening one stirrup by 5 cm, the fetlock extension at trot, which is a proxy for the peak vertical force, increased on the side contralateral to the shorter stirrup and altered the thoracolumbosacral range of motion [33].

Riders with asymmetrical foot pronation were found to have a more pronounced pelvic drop during weight bearing on the side contralateral to the foot with greater pronation (eversion) when walking in a straight line [34]. When the same riders rocked from side to side on an instrumented balance chair, pelvic and trunk roll were greater toward the side contralateral to the more pronated foot [35]. Furthermore, asymmetries recorded when rocking the balance chair were significantly related to asymmetries when riding; one degree of pelvis or head roll asymmetry on the chair predicted 2.4 degrees of asymmetry when riding [27]. The same asymmetrical posture was adopted whether the riders were riding on straight lines, circles, or leg yielding in the left or right direction. In relation to performance, the rider’s ability to move and control a Swiss ball with the pelvis was found to be a good predictor of harmony with the horse when riding [32]. A different group of riders were tested for their ability to perform anterior and posterior pelvic rotation on a Swiss ball. Recordings showed that they made compensatory movements of the lumbar spine comprised of leaning forwards or leaning backwards. All riders showed some compensatory movements when tilting the pelvis, and those with major compensations leaned further forwards, had greater left–right asymmetry and were more phase-shifted relative to the horse throughout the stride [33]. 

Collectively, these studies suggest there may be merit in assessing riders off horse, although a suitable battery of tests has not yet been defined. The asymmetries could then be addressed with therapy and specific exercises in an effort to limit their effects on the horse. The studies also highlight the importance of assessing the rider’s body as a whole rather than focusing on individual segments in isolation. 

Since both the horse and rider are likely to be inherently asymmetrical, it can be difficult to separate their relative contributions to an asymmetrical performance. Studies are needed to identify appropriate tests to pinpoint and distinguish left–right asymmetries in horses and riders and, further, to determine appropriate therapeutic exercises to restore a more symmetrical performance. Ideally, the rider should be dynamically stable, functionally symmetrical, and able to manage dynamic forces transmitted from the horse. 

## 3. Rider’s Posture and Movements

Horses’ gaits differ, among other things, in their limb coordination patterns and CoM trajectories. The rider’s goal is to follow the motion of the horse’s back primarily through controlled movements of the pelvis [19]. This implies suppleness of the hip joints, which avoids disturbing the leg position and lumbosacral joint, which maintains the vertical orientation of the axial body segments. Temporal synchronization of the rider’s movements with those of the horse is recognized as being a critical determinant in the appearance of harmony [36].

Rapid acceleration or deceleration of the horse tends to perturb the rider’s position due to the effects of inertia and momentum. If the horse stops suddenly (for example, when refusing a jump), the rider’s momentum carries the body forward and may result in a fall with the rider’s body continuing to move forward after the horse stops. Conversely, if a horse takes off to jump a stride earlier than expected, inertia causes the rider’s body to be ‘left behind’. If a rider can anticipate the horse’s direction of acceleration, the unbalancing effect can be reduced by leaning toward the anticipated movement. For example, a racehorse leaves the starting stalls in a forward direction, so it is appropriate to lean forward as the gate opens. On the other hand, a cutting horse follows the direction of movement of the steer, which is unpredictable, so a neutral position is adopted and the core musculature is engaged to avoid being thrown off balance when the horse moves. 

### 3.1. Rider’s Posture at Walk

At walk, the motion of the horse’s back and the magnitude of the GRFs impart relatively small perturbations to the rider, so the sitting position is used almost exclusively. The inverted pendulum mechanics of the horse’s limbs during walking result in the withers and croup being lowest at limb contact/lift off and highest at midstance of the forelimbs and hind limbs, respectively [37]. The out-of-phase rise and fall of the withers and croup causes the trunk to pitch nose-up in forelimb midstance (hind limb contact) and nose-down in hind limb midstance (forelimb contact) [38]. The middle of the back follows a smooth trajectory with minimal changes in height during the stride, and the total force exerted by the rider on the horse’s back is close to the rider’s weight throughout the stride (with only small variations) [15]. Longitudinal forces on the rider at walk are also small because as one limb provides a propulsive force, another limb provides a braking force. The combination of small vertical and longitudinal forces makes it easy for the rider to follow the horse’s back motion at walk.

In one study, the rider’s pelvis was reported to pitch in counter rotation to the saddle [39], but the rider’s trunk did not appear to have a clear pitching pattern. In contrast, Egenvall et al. [40] found that pelvic pitching movements in walk differed between riders both in amplitude and timing, with the rider’s pelvis pitching either in-phase or out-of-phase with the horse’s motion. Skilled riders maintain a greater posterior pelvic pitch throughout the walk stride, which is associated with greater nose-up pitch of the horse’s trunk, whereas novice riders had a greater anterior pitch with the pelvis displaced to the right [41]. Unskilled riders moved their pelvises in advance of the horse’s motion at walk [42], which may reflect using the seat as a pushing aid [43]. Taken together, these studies illustrate the possibility of different temporal coordination techniques being used to maintain the rider’s verticality in walk. High variability of relative phase values in walk points toward unstable phase coupling in this gait [42].

For the other axial rotations, the horse’s back has one complete roll cycle per walk stride in which it rolls toward the grounded hind limb, reversing the direction when the contralateral hind limb makes ground contact. The rider’s pelvis rolls in the same direction as the horse’s back [40] through a range of ~5° per stride [39,41].

There is a single yaw cycle of the horse’s back per walk stride with maximal left and right yaw occurring at contact of the left and right hind limbs, respectively [39]. The rider’s pelvis twists away from the grounded hind limb [39], while the rider’s trunk yaws in the opposite direction.

### 3.2. Rider’s Posture at Trot

When supported by a diagonal pair of limbs, the horse has a grounded hoof on each side of the CoM in longitudinal and transverse directions, which provides stability against pitch and roll rotations [44]. There are two pitching cycles per stride but the range of pitching rotation is only about 4° [45]. In the early diagonal stance, the trunk pitches nose-down as the CoM descends to its lowest point in the middle of diagonal stance under control of vertical and braking forces generated by the grounded fore and hind limbs. Later, in the diagonal stance phase, the GRFs propel the CoM forward and upward as the trunk pitches nose-up [46]. 

There are single cycles of roll rotation and yaw rotation in each trot stride [47] and their maxima coincide with the contact of the ipsilateral forelimb. Additionally, as the trotting speed increases, the horse’s abdominal and epaxial muscles activate earlier in the stride and with a greater amplitude to further stabilize the horse’s back [48], thus increasing the challenge of riding in harmony.

The effects of gravity and inertia on the cantilevered neck and head segments cause them to nod down slightly after the trunk reaches its lowest point, which the rider feels as an increase in rein tension just after midstance [49].

The total force exerted on the horse’s back has a sinusoidal pattern that increases in the first half of the diagonal stance, peaks just after midstance, and then decreases as the horse and rider rise [15]. The total force on the horse’s back summed over the entire stride is the impulse. It does not differ between seating styles, but the force is distributed differently through the stride cycle, resulting in differently shaped force–time curves [47].

The trot is, for most riders, the most difficult gait to sit due to the large vertical and longitudinal accelerations and decelerations produced by the synchronous contacts and movements of the diagonal limbs, the large range of motion of the horse’s back in the suspension phases, and the rapid (~3 Hz) repetitions of the oscillations [50]. The rider can avoid the challenges of sitting the trot by adopting different postures and kinematic patterns to follow the rhythmic movements of the horse’s back. These rider postures have been shown to require changes in the rider’s body and limb stiffness as well as damping functions [51]. The interaction between the horse and rider during trotting was studied using three models of force/damper/mass systems that included a period of freefall, dampers for the horse and rider, and an active spring system for the rider’s legs featuring variable stiffness and resting lengths. The output of the models showed which combinations of rider mass, spring stiffness, and damping coefficient were needed for the different rider postures [51].

#### 3.2.1. Sitting Trot

In sitting trot, the rider remains seated in the saddle throughout the stride. The horse’s back is highest around the start of suspension [45], but the rider continues briefly on an upwards trajectory that unweights the horse’s back [52]. The horse then descends under the influence of gravity, reaching its lowest point in the middle of diagonal stance, and is already starting to rise as the rider’s seat reaches its deepest position in the saddle. The rider’s downward momentum is reversed by a saddle reaction force that is approximately double the rider’s weight [15]. 

Rider kinematics follow a consistent pattern. As the horse’s trunk pitches nose-down early in the diagonal stance phase, the rider’s pelvis tilts anteriorly by 13–15° and the rider’s trunk pitches posteriorly by ~11° to resist the longitudinal decelerating forces [53]. This hollows the rider’s lumbar spine [54]. In the second half of the stance phase, the rider’s lumbar lordosis is flattened by a combination of the pelvis pitching posteriorly and the trunk anteriorly, which helps the rider to follow the horse’s acceleration.

As the rider descends, the seat presses forward and downward against the saddle, the legs slide forward as their joints flex, the toes move laterally, and the heels are lowered [54]. The head translates forward [54] to absorb the decelerative forces. Compared with pelvic movements, trunk movements are slightly delayed, and head movements are out-of-phase. 

The rider’s pelvis rolls in the same direction as the horse’s back but with a larger range of motion [54]. It rolls away from the grounded forelimb, with 4–5° range of motion [39,41,55]. In the second half of the diagonal stance phase, the pelvic roll varies in magnitude and direction, and may be asymmetrical to the left and right, perhaps reflecting the asymmetrical propulsive forces from the horse’s left and right hind limbs. The lateral bending and roll rotational movements of the horse’s cranial thoracic spine are reduced in the presence of a rider [56].

The horse, saddle, and rider’s pelvis yaw in the same direction, twisting away from the grounded hind limb until the contact of the next hind limb when the direction reverses. The range of yaw rotation is larger for the rider’s pelvis (~8°) than for the saddle (~6°) [39]. The rider’s trunk yaws in the opposite direction to the pelvis with 5–6° range of motion. 

The movements of expert riders are continuously phase-matched with those of the horse, whereas novices are less closely synchronized with the horse [36]. Coordinated movements of the rider’s shoulder and elbow joints throughout the stance phase maintain the position of the wrist relative to the bit within a range of <15 mm during each stride [57]. However, professional riders maintain more flexed elbows during sitting trot and have smaller ranges of flexion–extension in their knees [25].

As the rider is pushed upward, the horse’s back is unloaded. The movements that occurred during the first half of the stance are reversed, with the rider’s head and legs moving back and the leg joints extending [39].

Sitting trot exerts two symmetrical force peaks per stride on the horse’s back with the maximal force approximately twice the rider’s body weight [15]. The vertical force on the rider [52] and the stirrup forces [58] reach zero during the suspension phase at sitting trot, indicating that the saddle is unloaded, although this is not generally visible to an observer. 

The rider follows the horse’s movement by using a relatively high spring stiffness and a high damping coefficient [51]. The low stirrup forces are likely to limit the influence of the rider’s legs, which suggests that the lower back is the main determinant of the rider’s mechanical properties and is likely key to providing the high spring stiffness needed in sitting trot. However, a wide range of combinations of the rider’s spring stiffness and damping coefficients can result in sitting trot [51].

#### 3.2.2. Rising Trot

When performing rising trot, the rider sits in the saddle and rises out of the saddle on alternating diagonal steps. The simulation of rising trot using the spring/damper/mass model requires an active spring system for the rider’s leg, with the ability to change both its stiffness and resting length. Modeling of these conditions produced force patterns on the saddle and the stirrups, similar to those measured experimentally [52,58]. 

Pitch and roll rotations of the rider’s pelvis are similar in magnitude and direction to sitting trot [55]. The rider’s skill level affects muscle activation patterns and the associated energetic efficiency. Less skilled riders coactivate the rectus abdominis and iliocostalis lumborum, which is energetically expensive [59], whereas more skilled riders show a phase shift between these two muscles, with the rectus abdominis turning on from the time the rider contacts the saddle until the period of lumbosacral flexion [60]. 

Equitation texts indicate that, when riding through turns or on circles, the rider should sit when the outside forelimb and inside hind limb are grounded. This is because the asymmetrical distribution of the rider’s weight tends to counteract the circle-induced asymmetries in the horse’s weight distribution [61]. Conversely, if the rider sits on the inside forelimb and outside hind limb diagonal, the circle-induced asymmetries are magnified, and the horse may appear to be lame [61]. Due to the asymmetrical forces on the horse’s back in rising trot [62], it is recommended that the diagonal be changed frequently.

The saddle force at rising trot is characterized by a smaller force peak in the sitting phase and a larger force peak in the standing phase [47]. On the sitting diagonal, thoracolumbar extension is similar to sitting trot; however, on the rising diagonal, the measurements are more similar to the unloaded situation [62,63]. Forces on the rider during the sitting phase are dominated by the saddle spring; in the standing phase, the active spring system of the rider’s leg takes over. The incorporation of an active spring system for the rider’s leg was needed to simulate rising trot [51]. When the rider has low spring stiffness and low damping, both the horse’s workload and peak forces on the back decrease. 

During the sitting diagonal, there is a larger saddle contact area and higher mean pressure, especially under the middle and caudal parts of the saddle that are directly beneath the rider’s seat [64]. Left and right stirrup forces show 2 peaks per stride with higher values on the standing diagonal in association with high pressures beneath the stirrup bars [64]. 

Maximal croup height, which is normally symmetrical on the two diagonals, is lower during the rising phase, likely due to the downward momentum induced as the rider pushes against the stirrups to rise out of the saddle [57]. This mimics a push-off lameness in the hind limb of the sitting diagonal. Compared to trotting in hand, rising trot induced a measurable asymmetry of back movement during the weight-bearing and push-off phases of the stride [61].

#### 3.2.3. Standing Trot

In some equestrian sports, it may be advantageous for riders to shorten their stirrups and adopt a standing (two-point) position in which the rider’s weight acts through the stirrups. The horse’s vertical back motion is absorbed by flexion of the rider’s hip, knee, and ankle joints as the horse’s trunk rises into the suspension phases and then the extension of these joints as the horse descends in the diagonal stance phase. As a result of these joint movements, the rider’s body undergoes relatively small vertical and longitudinal oscillations that differentiate standing from rising trot accelerometrically [65]. 

The standing technique is the most demanding position for the rider and the muscular activity needed to coordinate movements of the trunk, arms, and legs has a high metabolic cost. This position requires a relatively low spring stiffness combined with a low damping coefficient [51]. It is important to control the stiffness in the rider’s legs because vertical displacement is very sensitive to a change in spring stiffness. There appears to be an optimal combination of the damping coefficient and spring stiffness; if the damping coefficient is too high, it increases the horse’s work. Alternatively, if the spring stiffness is too high, it increases the peak forces on the rider and on the horse’s back [51]. 

In standing trot, the horse moves symmetrically [61] and has symmetrical force maxima with lower values than those of sitting trot [66]. The minima, however, are relatively high because they occur during the phase when the rider pushes against the stirrups to raise the seat out of the saddle. Thus, the force–time curve has a flatter profile with the force distributed more evenly through the stride [47].

Riders often use rising trot as an easier alternative for the horse than sitting trot. However, standing trot has advantages over rising trot in terms of symmetrical pressure distribution on the two diagonals and lower peak vertical forces. Standing trot should be considered a good option for rehabilitation, starting young horses, and during warm-ups.

### 3.3. Rider’s Posture and Movements at Canter

In canter, the horse’s limb placements follow a caudal-to-cranial sequence with the footfalls alternating from side to side. The limb sequencing pattern of canter is associated with smaller longitudinal accelerations and decelerations than trot, which facilitates the rider’s task of following the movements of the horse’s back and makes canter an easier gait to sit than trot [42]. After the suspension phase, the horse’s trunk descends until the downward motion is decelerated sequentially by the trailing hind and the diagonal limb pair. The CoM reaches its lowest point during the diagonal stance after which it rises into the next suspension phase. The trunk has an uphill orientation at the start of the stance phase, and then pitches in a nose-down direction from 25 to 75% of the stance phase as the CoM descends. In the later part of stance, the trunk pitches in a nose-up direction [38]. The saddle reaction force required to reverse the rider’s descent into the saddle in canter is up to three times the rider’s weight [15].

Overall, higher levels of horse–rider synchronicity and coordination are present in canter compared with walk or trot, which supports anecdotal claims that canter is the easiest gait to sit [42]. Riders follow rather than anticipate the horse’s movements in canter, with a significantly shorter phase lag than in walk and trot, supporting the notion of a tighter rider–horse synchronization in canter [42]. 

Canter can be ridden in the same rider positions as trot: sitting, rising when the rider sits and stands on alternate strides, or standing. When sitting canter, the rider’s pelvis pitches out of phase with the horse’s trunk through a range of ~18° [25]. Initially, the movement of the rider’s CoM is directed toward the grounded trailing hind limb, then reverses direction towards the leading hind limb during diagonal stance, and finally rolls toward the leading forelimb [25]. The rider’s pelvis rolls in the opposite direction to the horse’s trunk with a range of ~6° [25], with skilled riders showing less pelvic roll than novices who tend to maintain the pelvis rolled to the right [25]. The rider’s trunk has more yaw rotation in canter than in the other gaits. In one study, the shoulders preferentially rotated to the left in all gaits except right lead canter [22]. 

The modern jockey technique with very short stirrups, marked flexion of the leg joints, and a horizontal torso exerts a more consistent vertical force with a lower peak force on the horse’s back [67]. The jockey undergoes a relatively small range of motion relative to the horse longitudinally [67], and the horse’s energy expenditure is reduced. After jockeys adopted this position, the race times decreased, and it was hypothesized that horses were able to gallop faster because the jockey became uncoupled from the horse, thus lowering the peak vertical forces [67].

## 4. Rider–Horse Coupling

The coupling between the horse and rider has been studied by a number of authors and using a variety of techniques. These include comparisons of the variations in timing of movement cycles within the rider [68] or between the horse and rider [25,41] using methods that were developed to analyze complex systems, such as phase plane diagrams [69] and approximate entropy [42], and methods developed to investigate the harmonics of motion [70]. The focus of these studies was principally to evaluate aspects of coordination variability, often with the motivation to understand and objectify skill and rider–horse harmony with the expectation that coordination will be more consistent in more skilled riders.

The rhythm of each gait and timing of the stride cycles adds to the complexity of the coupling between the horse and rider. In dressage, higher scores are partly attributed to maintaining the rhythm within each gait, despite increases in speed or changes in direction. In addition, a slow stride frequency in walk, trot, and canter is associated with good judged gait scores [14] and collective marks [71]. The goal of a skilled rider is therefore to coordinate their own motor behavior to the natural frequency of the horse [36,43]. This has been illustrated during steady-state trot [69], but the difference in the frequencies of gait patterns, speeds within gaits, and changes in direction can disrupt or alter the coordination patterns of the rider, which can then affect the horse’s rhythm. 

Changes in speed within gaits and changes in gait during simulated riding were found to induce alterations in pelvic movement strategies [68]. In this study, the frequency and magnitude of simulator oscillations were expected to alter the neuromuscular strategy required to maintain dynamic postural control. In another study, the rider–horse coupling was assessed by evaluating several coordination variability measures when circling left and right in walk, trot, and canter [42]. Surprisingly, no differences were found between left and right circles, but again, significant differences were found between gaits. A key finding was that more predictable sternal acceleration patterns were found in dressage riders at canter than at walk or trot, which was partly attributed at walk to having a faster, four-beat stride pattern [42].

The challenge in neuromotor control is to maintain a coordination pattern that resonates with the horse. The challenge increases in complexity when applying cues to change gait or direction. This was illustrated when investigating the frequency components of trunk to pelvis motion between straight trot and shoulder-in left and right [70]. In more skilled riders, greater symmetric frequency components were found when executing shoulder-in right compared to straight trot. This resulted in lower judged scores, suggesting a possible amplification of symmetric motion by the rider to apply cues for shoulder-in that disrupted the horse’s rhythm. Other factors may also influence the horse–rider coupling, such as saddle and riding style [72]. 

## 5. Conclusions

The posture, balance, and symmetry of the rider reflect their skill and experience in following the movements of the horse in different gaits. An experienced rider controls their weight distribution and body movements during predictable gait-related events and responds rapidly during unpredictable reactions of the horse. In order to accomplish this, the rider’s pelvis follows the movements of the horse’s back and the saddle, while the trunk stabilizes the upper body above the pelvis. Inherent and acquired asymmetries are common in both riders and horses and tend to be retained across gaits. There is evidence to indicate that it may be possible to identify and remedy rider asymmetries off horse. The ultimate goal is to enhance the harmony between the rider and the horse, which is the key to a successful biomechanical partnership between horses and riders.

With regard to the rider’s influence during equine rehabilitation, the presence of suspension phases in trot and canter is associated with greater vertical excursions of the horse’s body. An appropriate seating style should be chosen with consideration of the evidence presented here, which has shown that the two-point position has a more consistent loading on the horse’s back and avoids the high peak loads associated with sitting trot. 

## Data Availability

No new data were created or analyzed in this study. Data sharing is not applicable to this article.
